# PRaG Therapy of Refractory Metastatic Gastric Cancer: A Case Report

**DOI:** 10.3389/fimmu.2022.926740

**Published:** 2022-07-07

**Authors:** Hong Xu, Zhihui Hong, Meiling Xu, Yuehong Kong, Yifu Ma, Chanchan Shan, Pengfei Xing, Liyuan Zhang

**Affiliations:** ^1^ Department of Radiotherapy& Oncology, The Second Affiliated Hospital of Soochow University, Suzhou, China; ^2^ Department of Oncology, Changshu Hospital Affiliated to Soochow University, Suzhou, China; ^3^ Institution of Radiotherapy & Oncology, Soochow University, Suzhou, China; ^4^ Laboratory for Combined Radiotherapy and Immunotherapy of Cancer, The Second Affiliated Hospital of Soochow University, Suzhou, China; ^5^ Department of Nuclear medicine, The Second Affiliated Hospital of Soochow University, Suzhou, China

**Keywords:** immunotherapy, radiotherapy, GM-CSF, gastric cancer, pMMR, case report

## Abstract

Patients with metastatic gastric cancer had limited treatments and often had a somber prognosis, especially when patients were unable to tolerate high-intensity cytotoxic treatment due to poor physical condition or organ dysfunction after the failure of standard therapy. Here, we reported a metastatic and proficient mismatch repair (pMMR) gastric adenocarcinoma patient with the Eastern Cooperative Oncology Group (ECOG) performance status score of 2 associated with hypoproteinemia and fatigue, and poor appetite that was unable to tolerate high-intensity therapy after several chemotherapy regimens and anti-angiogenic therapy. After receiving novel triple-combination therapy, which consists of PD-1 inhibitor, Radiotherapy and Granulocyte-macrophage colony-stimulating factor (GM-CSF) therapy (PRaG for short), the patient achieved a complete response (CR) with a progression-free survival time of 14 months, and ECOG performance status score improved from 2 to 0. A significant systemic effect was observed in this case and the PRaG triple-combination therapy might provide a novel treatment strategy for metastatic pMMR gastric cancer patients.

## Introduction

In 2017, the PD-1 inhibitor pembrolizumab was approved by the US FDA to treat solid tumors with microsatellite high instability (MSI-H)/mismatch repair-deficient (dMMR). It was regrettable that dMMR/MSI-H only occurred in 2.2%-3.8% of solid cancers (1, 2). The incidence of dMMR/MSI-H in gastric adenocarcinoma was about 9%-21.9% (2–4); however, it was even lower in advanced gastric adenocarcinoma, with an incidence rate of less than 4% (3).In addition, immunotherapy was reported to have a response rate of only about 11% as monotherapy in gastric or gastro-oesophageal junction cancer refractory to or intolerant of standard therapy in the studies of KEYNOTE-059 and ATTRACTION-2 (5, 6).

It is essential to explore novel methods to sensitize immunotherapy, and the combination with hypofractionated radiotherapy (HFRT) or stereotactic body radiation therapy (SBRT) may be a promising strategy. Here, we presented a case of a refractory metastatic gastric adenocarcinoma patient with pMMR who received a notable response after treatment of triple-combination therapy of PD-1 inhibitor combined with radiotherapy and granulocyte-macrophage colony-stimulating factor (GM-CSF).

## Case Presentation

**Figure 1:**
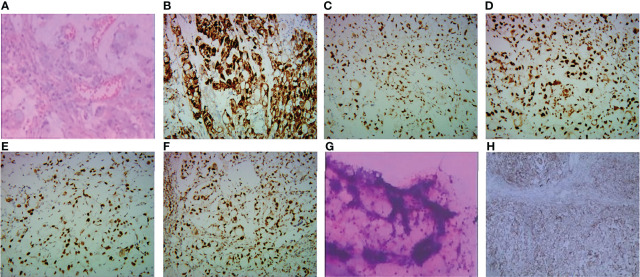
**(A–F, H)** Pathological and immunohistochemical staining of gastric cancer biopsy specimens from this patient. **(A)** Gastric adenocarcinoma (hematoxylin and eosin). **(B)** The tumor cells were strongly positive (3+) for HER2/neu immunostain. The expression of MLHI **(C)**, MSH2 **(D)**, MSH6 **(E)**, and PMS2 **(F)** was preserved in tumor cells. **(G)**There were no tumor cells in the pelvic effusion. **(H)** showed positivity for PD-LI CPS 60.

On 31 March 2020, a 67-year-old man was diagnosed with gastric cancer and underwent laparoscopic radical gastrectomy in the Second Hospital of Soochow University. Postoperative pathology revealed gastric adenocarcinoma with regional lymph nodes metastasis, pT3N3bM0 (p-Stage IIIC), with immunohistochemistry (IHC) HER2 (3+), pMMR (MLH1 (+), MSH2 (+), MSH6 (+), PMS2 (+) (**Figures 1A–F**). Subsequently, between 13 May 2020 and 25 July 2020, the patient was enrolled in a prospective phase I/II clinical trial for patients with local advanced gastric cancer (ChiCTR1900021702, http://www.chictr.org.cn/index.aspx) and received adjuvant radiotherapy combined with concurrent of two cycles of raltitrexed chemotherapy, followed by 2 cycles of oxaliplatin plus raltitrexed chemotherapy. Unfortunately, the carcinoembryonic antigen (CEA) was increased one month after the adjuvant chemotherapy, and multiple newly metastatic mediastinal lymph nodes were detected by CT scan on 26 August 2020, which was assessed as progressive disease (PD) and suggested that the tumor had high malignant behavior and was insensitive to first-line chemotherapy agents. The patient developed nausea, vomiting and thrombocytopenia with a poor condition and refused intravenous chemotherapy. And he received tegafur-gimeracil-oteracil potassium capsules and apatinib, but unfortunately, the patient developed severe oral and nasal ulcers during the treatment. However, after two months, CT scans showed significant enlargement of mediastinal lymph nodes and multifold increase of CEA level with pelvic effusion, and the patient was assessed as PD again. We then conducted an ultrasound puncture of the pelvic effusion, but no tumor cells were found in the effusion (**Figure 1G**). Immunohistochemical staining of the tumor tissue showed that the PD-L1 combined positive score (CPS) 60 (**Figure 1H**).

The patient presented with ECOG performance status score of 2, accompanied by fatigue, anorexia, and hypoproteinemia. And he was considered to be intolerant to further chemotherapy. Considering the above conditions, the treatment options were quite limited. The patient was then treated with PRaG therapy from November 2020 (7). The patient was treated with subcutaneous GM-CSF (200 µg once daily, d4-17) after the completion of radiotherapy (15Gy/3f, d1-3) and received a PD-1 inhibitor (toripalimab 240mg, d4).

**Figure 2:**
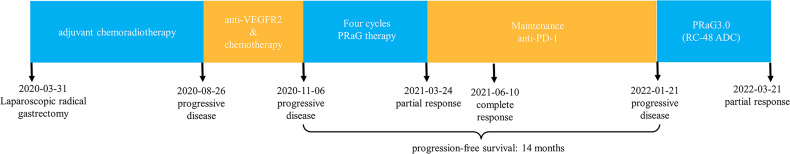
Timeline of the whole treatment process for the patient. The patient experienced PD with the new emergence of enlarged paratracheal lymph nodes during postoperative adjuvant chemotherapy on 26 August 2020. Two months after apatinib was combined with chemotherapy, the subcarinal LN, right upper paratracheal LN, and left lower paratracheal LN were detected by CT scan on 06 November 2020, and the patient developed PI) again, After 4 cycles of PRaG therapy, the sum of diameters of the unirradiated target metastases decreased by 45% from baseline, and the right upper paratracheal LN almost disappeared. The patient was assessed as PR on 24 March 2021.18F FDG PET-CT on 10 June 2021, most LNs disappeared and one shrunk to normal size with normal FDG uptake. Therefore the patient achieved CR. Although the patient unfortunately suffered PD again on 21 January 2022 (new metastatic LNs appeared), he obtained 14 months of PFS from the start of PRaG therapy on 19 November 2020. PRaG therapy: The patient was treated with subcutaneous GM-CSF (200 gg once daily, d4-17) after the completion of radiotherapy (15Gy/3f, d1-3) and received a PD-I inhibitor (toripalimab 240mg, d4.The PRaG regimen was repeated every three weeks, and different mediastinal lymph nodes were irradiated during the PRaG treatment, with each cycle delivered to one metastatic site. After four cycles of PRaG therapy, maintenance treatment ofPD-1 monotherapy was administered every three weeks. Until 21 January 2022, when the patient suffered PD again, Each cycle of the above has a duration of 21 days. LN, lymph node; PD, progressive disease; PR, partial response; CR, complete response; PFS, progression-free survival.

**Figure 3:**
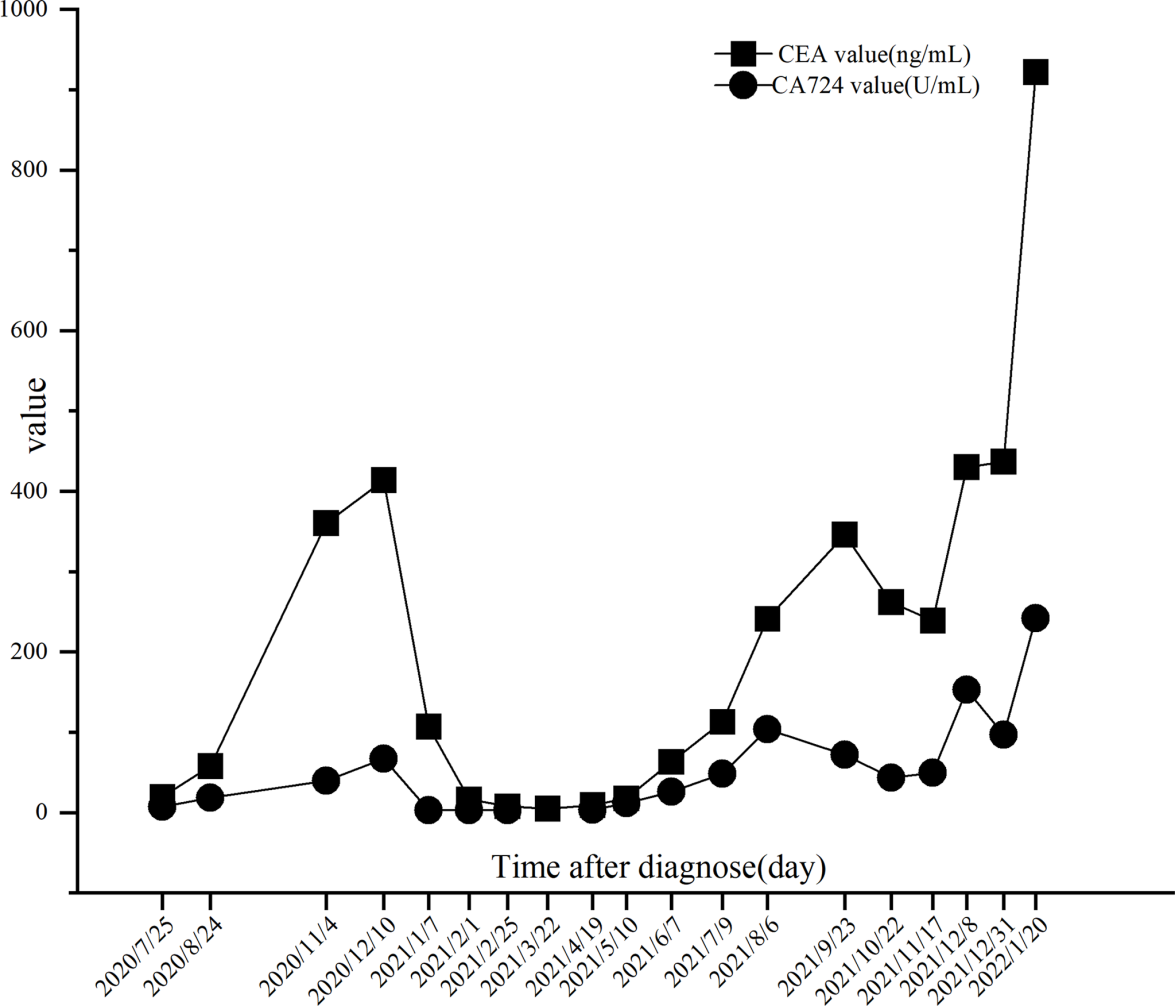
Trends of CEA and CA724 levels. The CA724 level was reduced to normal on 07 January 2021 after 2 cycles of PRaG therapy. The CEA level was decreased to normal on 22 March 2021 after 4 cycles of PRaG therapy. There was a fluctuating increase in both biomarkers from 6 August 2021 to 3 1 December 2021, but the disease remained stable after CT evaluation. On 20 January 2022, a multifold increase in the two biomarkers was detected, at which time the patient was assessed as progressive disease (PD) by CT scans.

**Figure 4:**
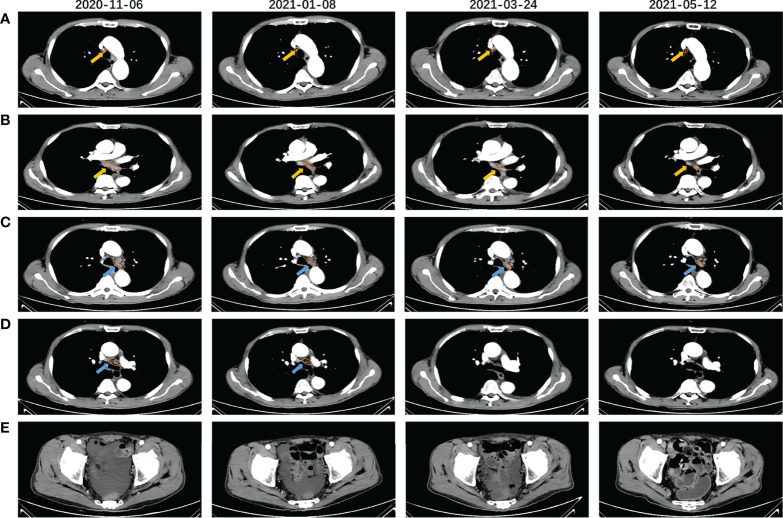
CT scans before (2020-11.06), during (2021-01-08), and after (2121-03-24, 2021-05-12) the PRaG therapy. Right upper paratracheal **(A)** and subcarinal lymph node **(B)** were the two irradiated lesions that gradually shrunk significantly after the PRaG therapy, One of the left lower paratracheal lymph nodes **(C)** progressively shrunk and the other **(D)** rapidly shrunk and disappeared after PRaG therapy. **(E)** shows that pelvic effusion gradually decreased and disappeared, The yellow arrow indicated the irradiated lesions, and the blue arrow indicated the unirradiated ones; all assessed lesions were circled with orange lines.

The PRaG regimen was repeated every three weeks and different mediastinal lymph nodes were irradiated during the PRaG treatment with each cycle delivered to one metastatic site. The patient was assessed as partial response with the Response Evaluation Criteria in Solid Tumors (RECIST v1.1) by CT on 24 March 2021. After that, maintenance treatment of PD-1 monotherapy was administered every three weeks. The patient got complete response on 10 June 2021 (**Figure 2**). Serum CEA and CA724 levels were measured every cycle (**Figure 3**)and an imaging assessment was performed every two cycles. Both irradiated and unirradiated sites got progressive shrinkage during the treatment (**Figures 4A–D**).

PET-CT scans were evaluated during the maintenance therapy on 10 June 2021, complete response (CR) with the RECIST v1.1 was assessed (**Supplementary Figures 1A–C**). The progression-free survival (PFS) was 14 months. The ECOG performance status score changed from 2 to 0, with increased appetite, improved hypoproteinemia, and diminished pelvic effusion (**Figure 4E**). The patient developed grade 1 fever and grade 2 pruritus according to Common Terminology Criteria for Adverse Events (version 5.0), which were considered to be associated with GM-CSF. The patient’s fever disappeared, and the pruritus was relieved in the absence of GM-CSF administration. Fever can be controlled with oral celecoxib (200mg, BID) and pruritus can be alleviated with oral loratadine (10mg, QD) and montelukast (10mg, QN). No grade 3 or higher treatment-related adverse events (TRAEs) occurred in this patient. TRAEs of any grade, such as thyroid and liver dysfunction, were not detected. After disease progression on 22 January 2022, the patient received RC-48 antibody-drug conjugate (ADC) combined with PD-1 inhibitor and radiotherapy sequential GM-CSF and interleukin-2 (IL-2) (PRaG3.0, NCT05115500). Excitingly, after only two treatment cycles, the patient was assessed as PR again on 21 March 2022 and treatment is still ongoing.

## Discussion

The treatment of advanced gastric cancer patients with poor performance status after the failure of multiple therapies was very tricky. The case we reported here was an advanced gastric cancer with HER2-positive and proficient mismatch repair(pMMR). The patient developed disease progression during postoperative adjuvant chemotherapy and rapidly progressed after tegafur-gimeracil-oteracil potassium capsules combined with apatinib, which was considered insensitive to chemotherapy and anti-angiogenesis therapy, and have a high degree of malignancy. The prognosis might be inferior, with overall survival (OS) of fewer than six months if the best supportive care was given alone (8, 9). After the above treatment, the patient’s physical condition deteriorated progressively, indicating that high-intensity chemotherapy agents were not tolerated. The PRaG therapy of PD-1 inhibitor combined with radiotherapy and GM-CSF was then selected.

The range of irradiated lesions was small, and the irradiation dose of each cycle was only15Gy in three fractions, which was far lower than the conventional radical irradiation dose (**Supplementary Figures 2A–D**). Fortunately, the patient obtained a best response and a durable remission and was well tolerated without noticeable adverse effects. The patient maintained a good physical condition with an ECOG score of 0 until the disease progressed in January 2022.

For HER2-positive metastatic/advanced gastric cancer, trastuzumab combined with chemotherapy should be used as first-line treatment. The combination of pembrolizumab with trastuzumab and chemotherapy emerged as a new first-line treatment option for these patients based on the KEYNOTE 811 study (10, 11). For this patient, trastuzumab was refused for personal financial reasons, and pembrolizumab and nivolumab were approved for back-line treatment at that time. Subsequent therapies for this patient include chemotherapy such as paclitaxel and irinotecan if his condition is allowed, and anti-angiogenesis therapy such as ramucirumab and ADC. Median OS was 3.8 to 6.4 months with chemotherapy (12, 13) and 5.2 to 6.5 months with anti-angiogenesis therapy (14, 15). ADC of trastuzumab deruxtecan (DS-8201) had a favorable anti-tumor response with a median OS of 12.5 months but was not approved in China (16).

Pembrolizumab was approved in 2017 for the third-line or above treatment of advanced gastric cancer with PD-L1 CPS ≥1 based on the KEYNOTE 059 study (6), marking the advent of the era of immunotherapy for gastric cancer. Unfortunately, the indication was withdrawn due to the failure of OS benefit in KEYNOTE 061 and KEYNOTE 062 studies (17, 18). Pembrolizumab monotherapy was currently approved only for the advanced gastric cancer patients with MSI-H/dMMR) (3, 19)or high tumor mutation burden (TMB-H) (20)in the second-line treatment. Nivolumab for patients with advanced gastric cancer had a median OS of 5.26 months in the third-line treatment or beyond (5). Toripalimab showed a median OS of 4.8 months in chemo-refractory advanced gastric cancer (21). Immunotherapy provided more treatment opportunities for advanced gastric cancer, but monotherapy had not yet led to survival breakthroughs.

To explore strategies to improve the effectiveness of immunotherapy, we tried PD-1 inhibitor combined with radiotherapy and GM-CSF. In this case, the gastric cancer patient experienced multiple treatment failures and fortunately achieved a response of CR with PFS of 14 months after receiving PRaG therapy, although he was considered to have a low response to immunotherapy because of his pMMR status. None of the patients treated with toripalimab achieved CR in the Phase Ib/II trial NCT02915432 (21), which further reinforced our view that the synergistic effect of this combination therapy was associated with this favorable response.

High PD-L1 expression was considered to predict a better response to immunotherapy in specific tumors such as lung cancer. However, the predictive value of PD-L1 for gastric cancer needs further confirmation. Pembrolizumab showed no benefit of OS compared with chemotherapy in patients with recurrent or metastatic gastric cancer with PD-L1 CPS ≥1 in KEYNOTE 061 and KEYNOTE 062 studies (17, 18). The anti-tumor response or OS with nivolumab was not associated with PD-L1 status in the ATTRACTION-2 and ATTRACTION-4 studies (5, 22). The PD-1 inhibitor toripalimab showed no survival benefit in chemo-refractory advanced gastric cancer patients with PD-L1 overexpression than PD-L1 negative (21), indicating that this patient’s PD-L1 CPS 60 might not be associated with the excellent response to immunotherapy. In contrast, the synergistic anti-tumor immune effect between radiotherapy, GM-CSF, and PD-1 inhibitor was considered to play an essential role in response to immunotherapy.

Radiotherapy can initiate an anti-tumor immune response by inducing immunogenic cell death and efficiently releasing tumor antigens, and was increasingly being found to sensitize immunotherapy (23). Multisite SBRT could optimize PD-1/PD-L1inhibitors response by significantly reducing the overall tumor burden and promoting antigen release and presentation (24). Dendritic cells (DCs) are the principal antigen-presenting cells, which play an essential role in radiotherapy’s *in situ* vaccine effect and enhance immune checkpoint inhibitors (25, 26). GM-CSF is a cytokine that can regulate DCs differentiation, proliferation and survival (27), generate mature DCs such as CD8a^-^ and CD11b^+^ DC (28, 29), and stimulate an increase in the level of B7-1 at the immune site of the recruited DCs (28, 29), which was considered a promising approach in cancer immunotherapy (30).

Synergies between PD-1 inhibitors in combination with radiotherapy or GM-CSF have been shown in several clinical studies) (31–37). Theelen WSME et al. conducted a pooled analysis of PEMBRO-RT (33) and MDACC (38) trials and showed that the addition of radiotherapy to pembrolizumab significantly improved response and outcomes (PFS: 9.0 vs. 4.4 months, median OS: 19.2 vs. 8.7 months) in patients with non-small cell lung cancer (39). GM-CSF in combination with ipilimumab achieved a longer OS and lower toxicity than that ipilimumab alone in patients with unresectable stage III or IV melanoma (36). A proof-of-principle reported by Golden et al. showed that 26.8% of patients with metastatic solid tumors achieved abscopal responses after radiotherapy combined with GM-CSF, and these patients with abscopal responses had better OS (20.98 vs. 8.33 months) (37).

To our knowledge, the triple-combination therapy of PRaG was the first to be reported in gastric cancer. Ni J et al. reported the safety of the combination therapy of PD-1 inhibitor plus stereotactic body radiotherapy and GM-CSF for one cycle in a Phase II clinical study of advanced non-small cell lung cancer in September 2021, where fatigue, fever and bone pain were the most common treatment-related side effects (40). In our case, the main adverse events were fever and pruritus, considered GM-CSF related. Symptoms were relieved by oral medication alone.

There were also some deficiencies in the patient’s treatment, such as not receiving trastuzumab and ADC. Nevertheless, PRaG therapy achieved an exciting anti-tumor response and extended survival in this patient when no alternative treatment was available. The patient then participated in an ongoing clinical trial of ADC combined with PD-1 inhibitor and radiotherapy sequential GM-CSF and IL-2 in advanced solid tumors patients with HER2-expressing (PRaG3.0, NCT05115500). Excitingly, after only two treatment cycles, the patient was assessed as PR again on 21 March 2022, with an ECOG score of 1(**Supplementary Figures 2E**, **3**).

In summary, this case showed a significant response to PRaG therapy and clinical trials are needed to verify the efficacy and mechanism of this strategy.

## Data Availability Statement

The original contributions presented in the study are included in the article/**Supplementary Material**. Further inquiries can be directed to the corresponding authors.

## Ethics Statement

The studies involving human participants were reviewed and approved by Ethics Committee of the Second Affiliated Hospital of Soochow University. The patients/participants provided their written informed consent to participate in this study. Written informed consent was obtained from the individual(s) for the publication of any potentially identifiable images or data included in this article.

## Author Contributions

HX and ZH composed the manuscript, contributed equally to this work and shared the first authorship. LZ and PX designed and conducted the study as corresponding authors. MX and YK helped with data collection and interpretation. YM and CS collected and sorted out part of the image data. All authors contributed to the article and approved the submitted version.

## Fundings

This study was supported by the National Natural Science Foundation of China(82171828), the Key R & D plan of Jiangsu Province (Social Development, BE2021652), Suzhou Radiotherapy Clinical Medical Center (Szlcyxzx202103), Open project of the State Key Laboratory of Radiology and Radiation Protection of Soochow University (GZK1202014), Open Project of Provincial Key Laboratory of Soochow University (KJS1961), the Subject construction support project of the Second Affiliated Hospital of Soochow University (XKTJ-RC202001, XKTJHRC20210011), the Suzhou Science and Technology Development Plan (SYS2020143), Chinese Society of Clinical Oncology Research Foundation of Beijing (Y-XD202002/ZB-0015), Wu Jieping Medical Foundation (320.6750.2021-01-12), Open project of Provincial Key Laboratory of Soochow University (KJS1961), Suzhou Science and Education Health Project(KJXW2021018), Postgraduate Research & Practice Innovation Program of Jiangsu Provinc (SJCX22_1508), and the Subject construction support project of the Second Affiliated Hospital of Soochow University (the Talent support project of the Academy of Science and Education) [No. XKTJ-RC202015].

## Conflict of Interest

The authors declare that the research was conducted without any commercial or financial relationships that could be construed as a potential conflict of interest.

## Publisher’s Note

All claims expressed in this article are solely those of the authors and do not necessarily represent those of their affiliated organizations, or those of the publisher, the editors and the reviewers. Any product that may be evaluated in this article, or claim that may be made by its manufacturer, is not guaranteed or endorsed by the publisher.
