# Supplementary figures and images for: PRaG Therapy of Refractory Metastatic Gastric Cancer: A Case Report
Source: Front Immunol. 2022 Jul 7;13:926740. doi: 10.3389/fimmu.2022.926740 (PMC9300850; doi:10.3389/fimmu.2022.926740)

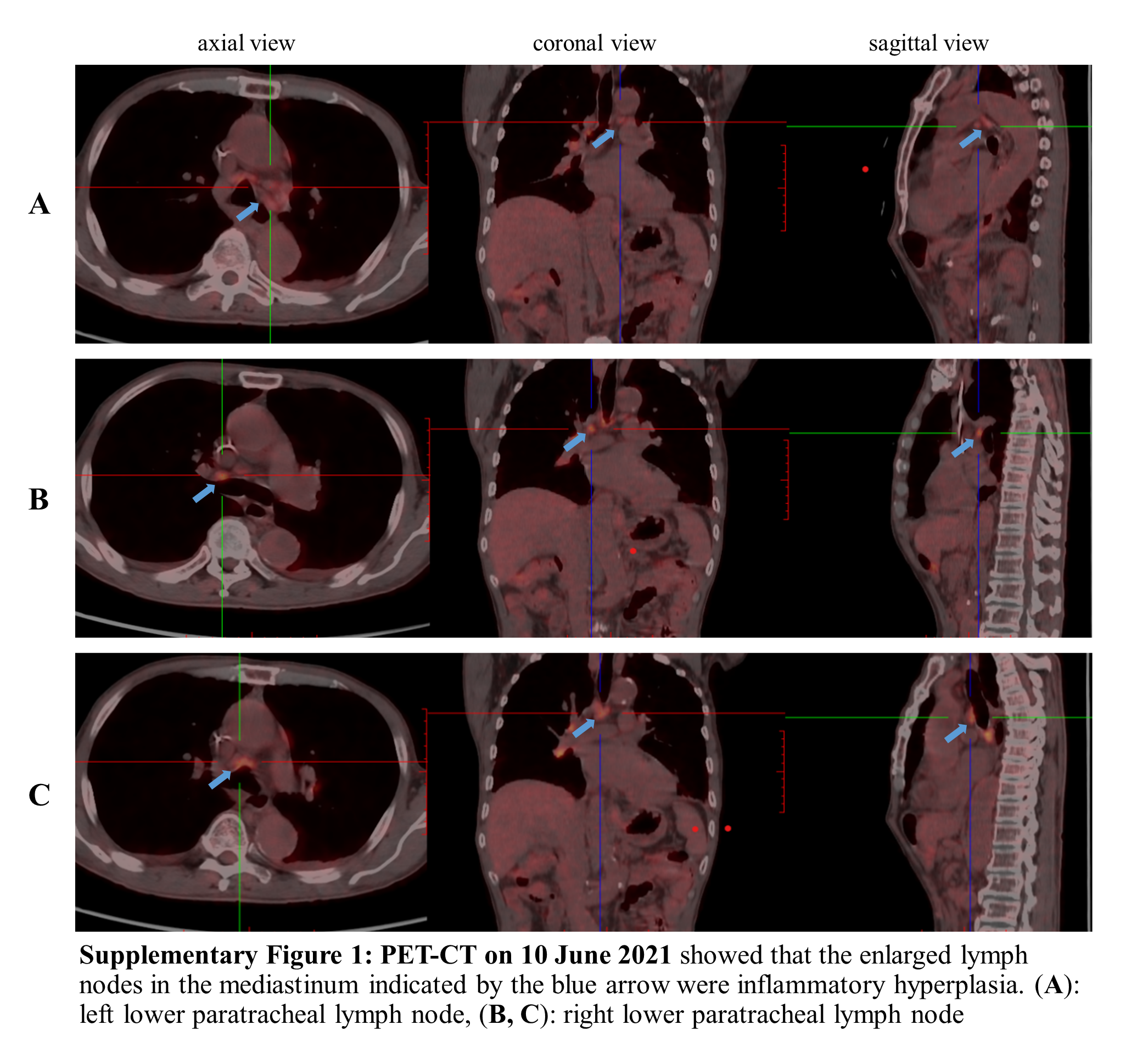

Supplement: Supplementary file 1 [file Image_1.tif]

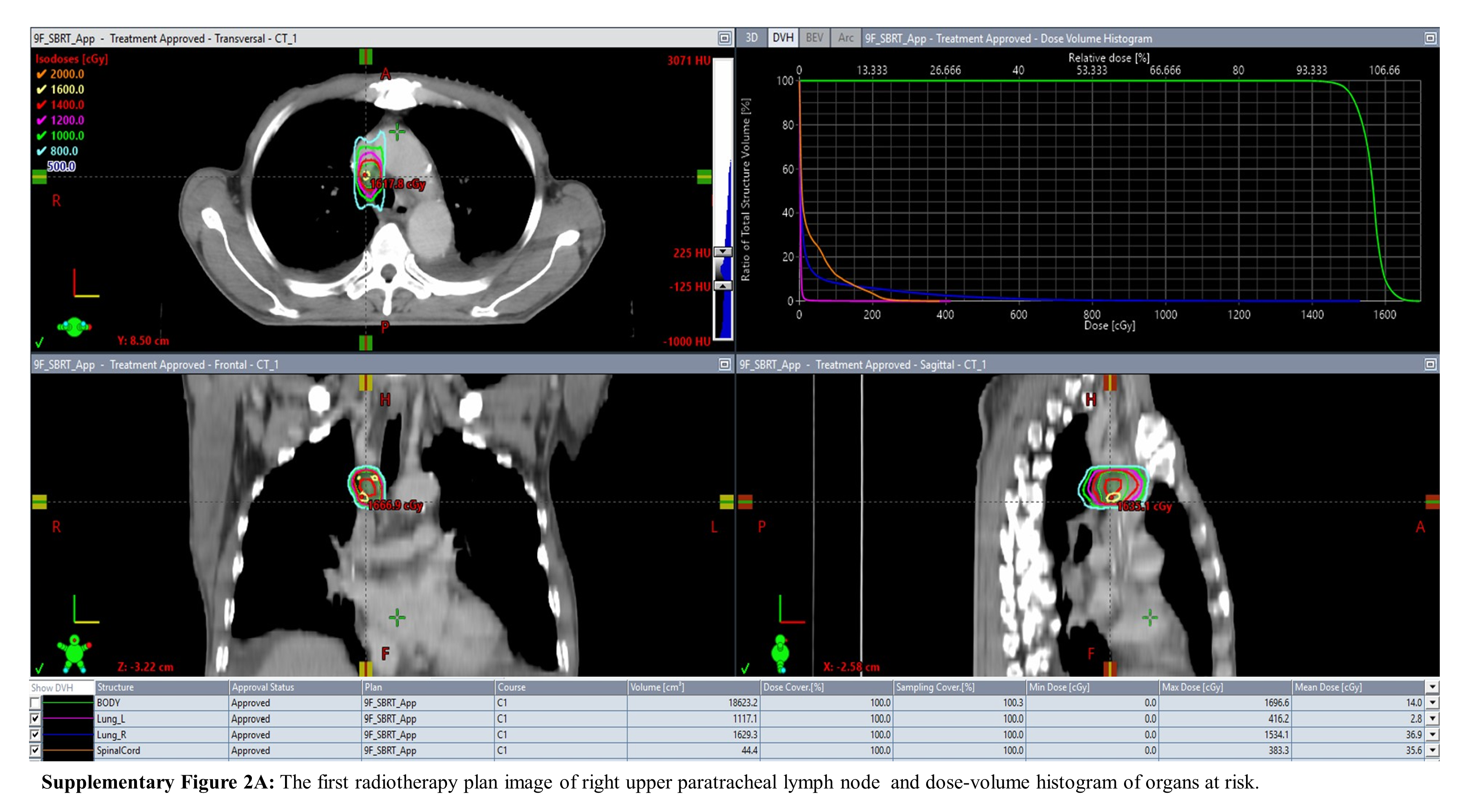

Supplement: Supplementary file 2 [file Image_2.tif]

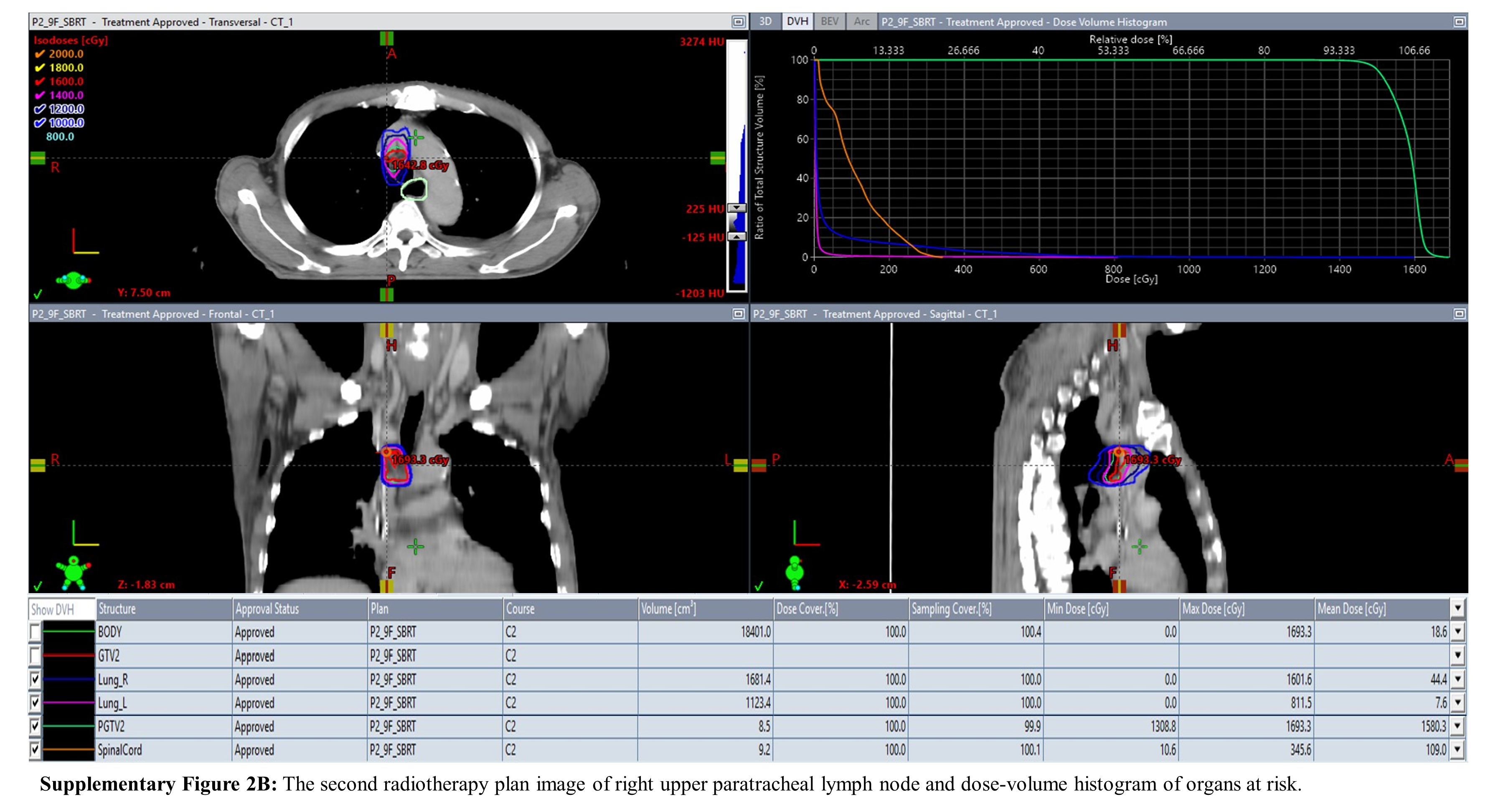

Supplement: Supplementary file 3 [file Image_3.tif]

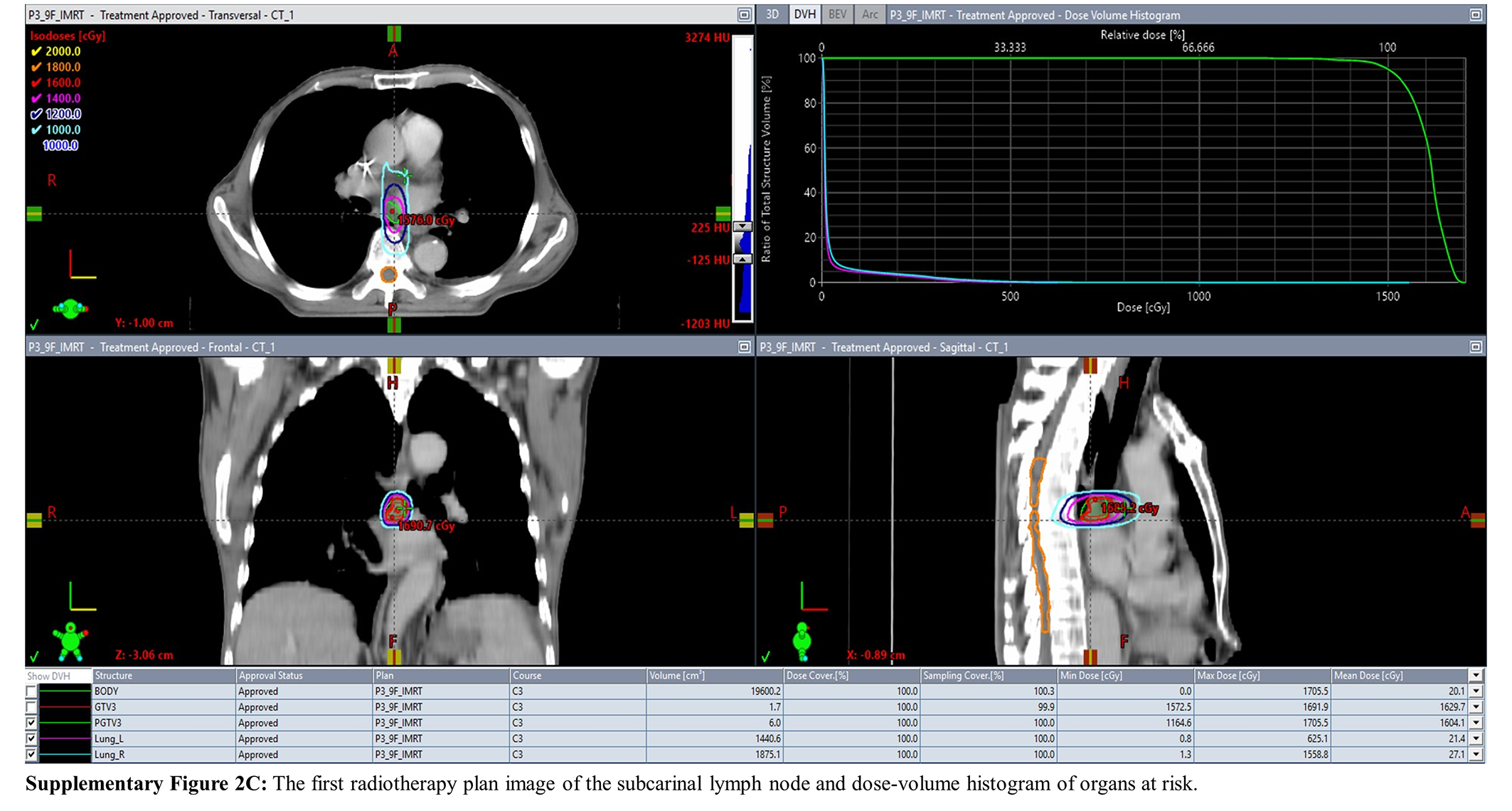

Supplement: Supplementary file 4 [file Image_4.tif]

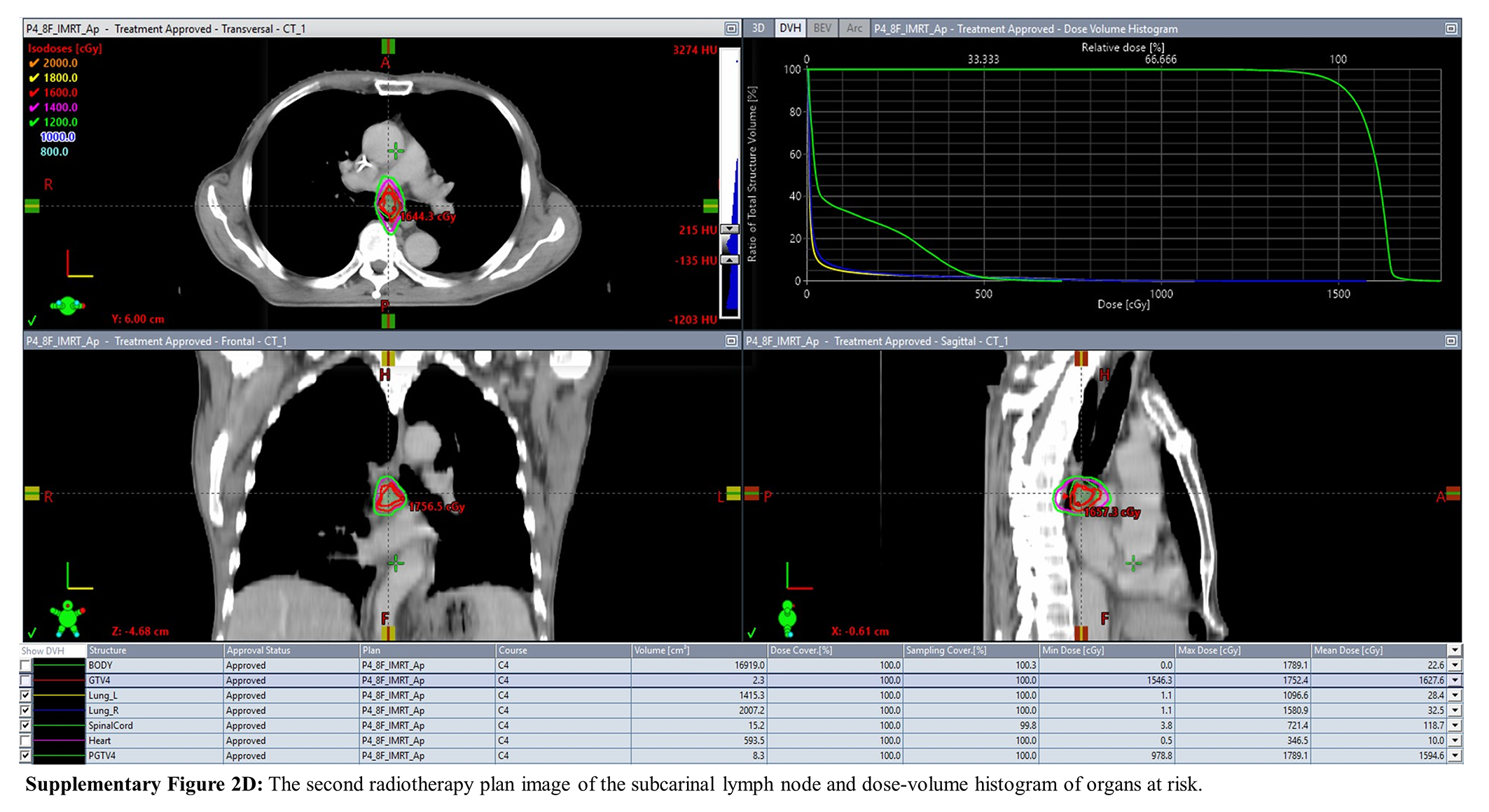

Supplement: Supplementary file 5 [file Image_5.tif]

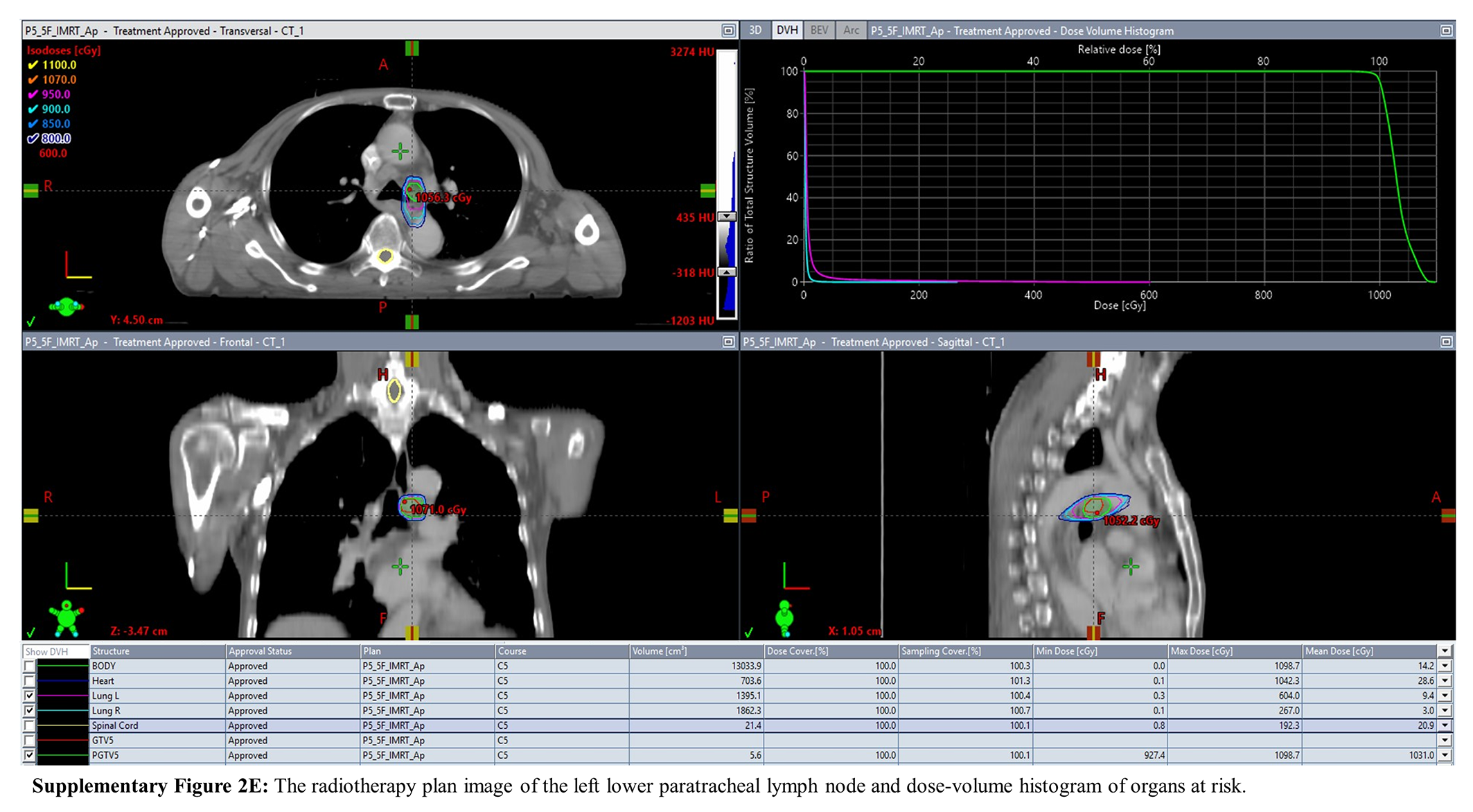

Supplement: Supplementary file 6 [file Image_6.tif]

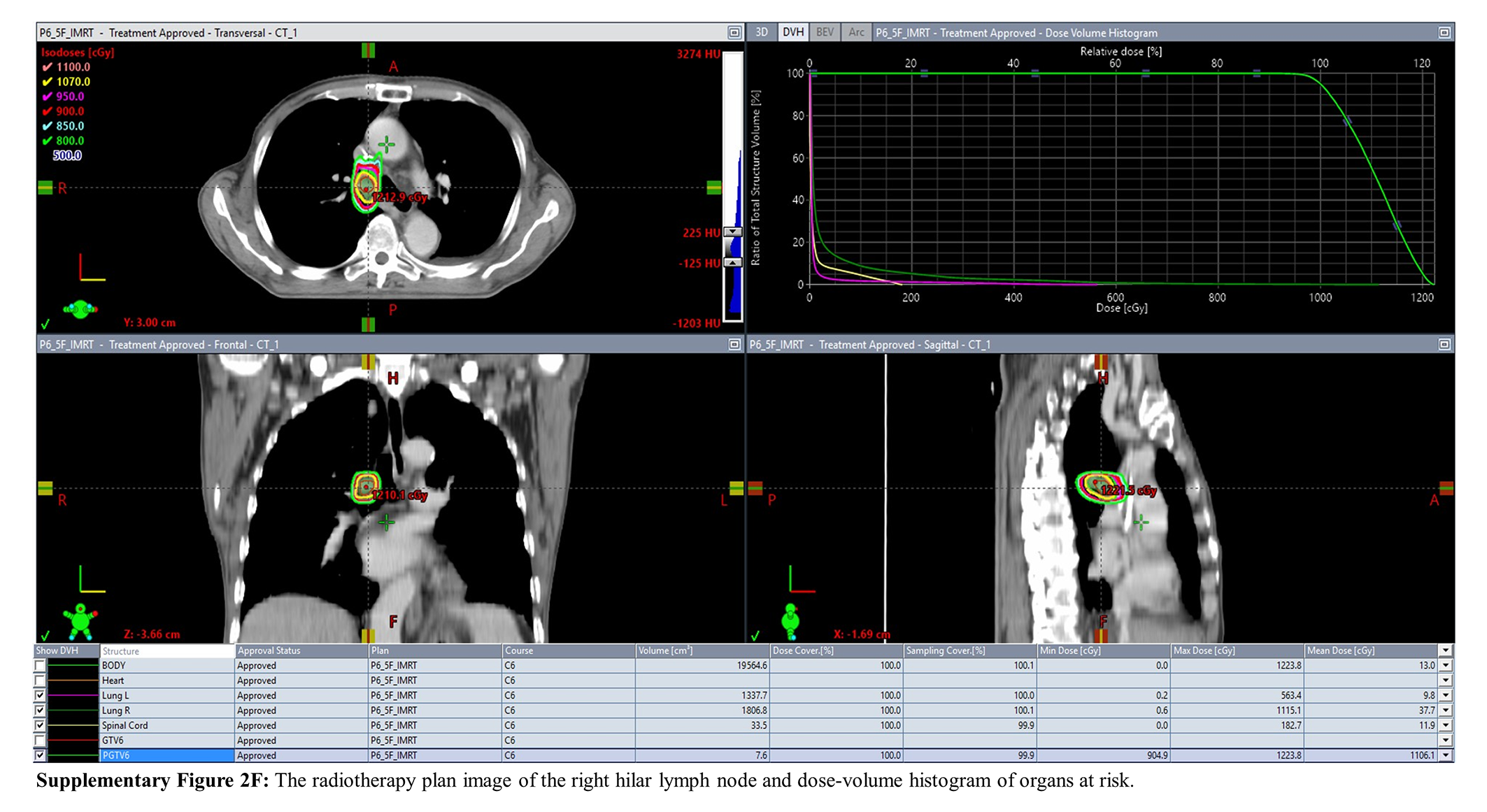

Supplement: Supplementary file 7 [file Image_7.tif]

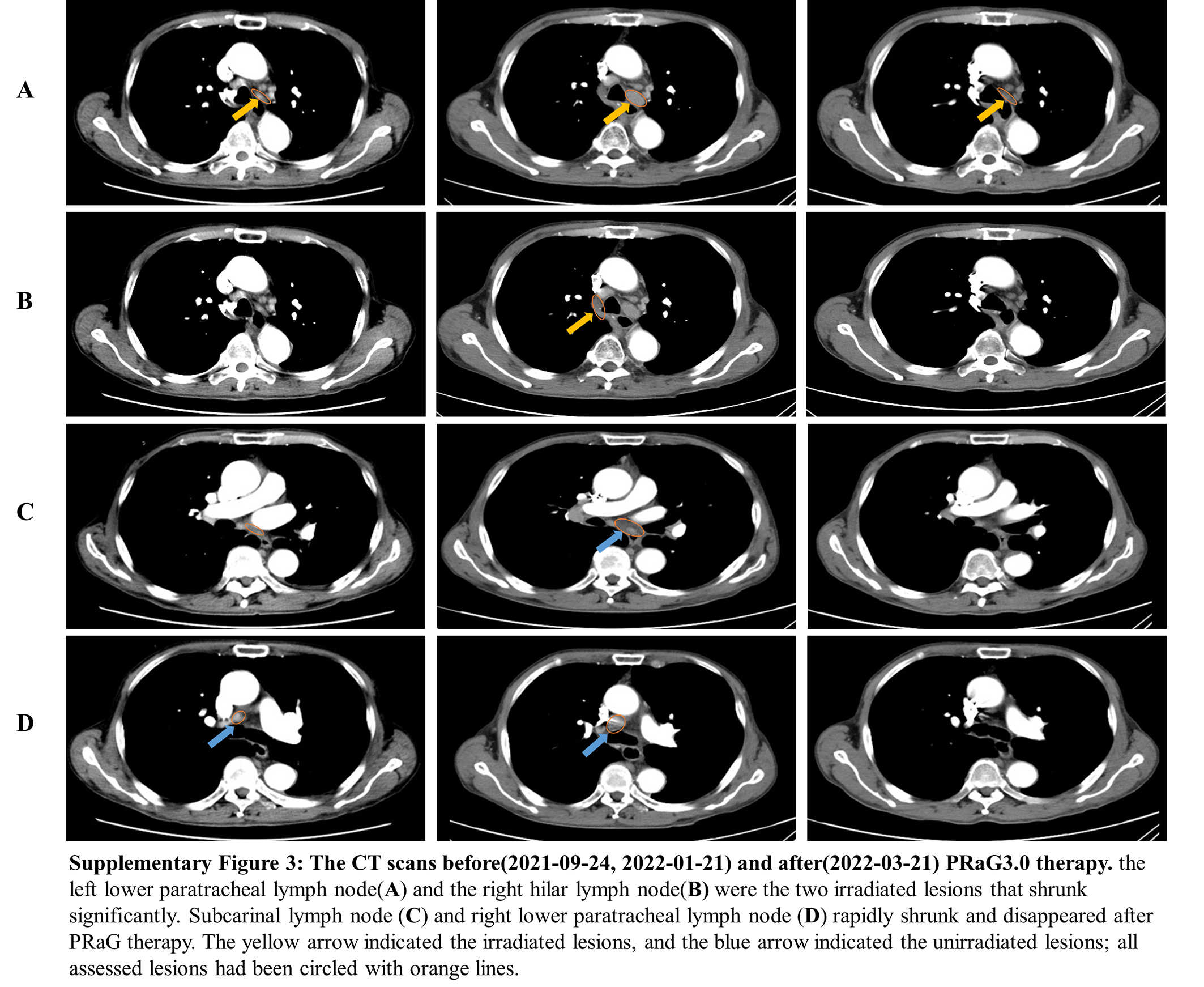

Supplement: Supplementary file 8 [file Image_8.tif]
